# Supplementary material for: Are carbon emissions trading and green financial instruments synergistic? -Comprehensive quantitative research based on content analysis
Source: PLoS One. 2024 Mar 7;19(3):e0298601. doi: 10.1371/journal.pone.0298601 (PMC10919601; doi:10.1371/journal.pone.0298601)
Supplement: S1 Data — (ZIP) [file pone.0298601.s001.zip › Data/地级市Z_F工作报告.zip!/║╙─╧/╓▄┐┌╩╨/└┤╫╘╣1⁄2╓┌║┼í╛┬φ┐╦╩2╛▌═°í┐_2015.docx]

各位代表：

　　现在，我代表市人民政府，向大会报告工作，请予审议，并请各位政协委员和其他列席人士提出意见。

　　一、2014年工作回顾

　　过去的一年，面对复杂多变的宏观经济环境和艰巨繁重的改革发展任务，在省委、省政府和市委的正确领导下，我们认真贯彻党的十八大和十八届三中、四中全会精神，深入实施周口崛起方略，牢记使命，务实重干，较好地完成了市三届人大五次会议确定的各项任务，经济运行稳中有进，各项事业稳步发展，人民生活持续改善，社会大局和谐稳定，在全面建成小康社会的征程中迈出了新步伐，取得了新成绩。

　　初步核算，全市生产总值1992.1亿元，同比增长9.1%，高于全省0.2个百分点；固定资产投资1375.4亿元，增长19.4%，高于全省0.2个百分点；规模以上工业增加值790亿元，增长12.3%，高于全省1.1个百分点；公共财政预算收入91亿元，增长19.6%，高于全省6.2个百分点；社会消费品零售总额852.9亿元，增长12.7%；城镇居民人均可支配收入19742.4元，实际增长8.5%，高于全省1.7个百分点；农民人均纯收入7741.8元，实际增长9.5%，高于全省0.1个百分点；节能减排目标全面完成。

　　特别值得欣慰的是，一些重点工作和重点项目取得了实质性进展，实现了历史性突破。

　　——主要经济指标高于全省平均增长速度。经济发展保持了较好的增长态势，尤其是生产总值增长速度已连续三年超过全省平均水平，经济总量稳居全省第5位。

　　——粮食生产再创历史新高。在抗御60多年来最严重干旱的情况下，粮食生产实现“十一连增”，全年粮食总产达到161.3亿斤，再创历史最好水平，稳居全省第1位，为国家粮食安全作出了新的贡献，我市再次被评为全国粮食生产先进市，7个县市被评为全国粮食生产先进县。

　　——地方财政总收入突破百亿元大关。地方财政总收入完成116.8亿元，增长18.1%。公共财政预算收入增速居全省第1位，提前一年超额完成“十二五”规划确定的“到‘十二五’末地方财政一般预算收入突破80亿元”的奋斗目标。

　　——郑合高铁即将正式开工建设。郑州经周口至合肥高铁已基本完成线路勘察、站点设置预可研等前期工作，预计下半年正式开工建设，周口即将结束没有高速铁路的历史，“大交通”体系更加完善。

　　——通往上海等大中城市旅客列车顺利开行。在已开行通往北京、广州直达列车的基础上，成功增开通达上海、天津、重庆、青岛、南通、襄阳等城市的旅客列车，并将广州返程列车延伸到周口，大大方便了我市群众出行。

　　——隆达大型电厂建设取得实质性进展。投资53亿元的隆达2×66万千瓦燃煤电厂项目前期工作全面完成，2014年10月正式试桩建设，该项目建成投产后，将为我市今后一个时期的发展提供强大的能源支撑。

　　——新周高速公路建设快速推进。郑州新郑国际机场到周口的高速公路土方、桥涵等基础设施建设已基本完成，今年年底建成通车后，更有利于周口融入郑州航空港经济综合试验区和全省核心经济圈。

　　——华耀商贸城一期工程建成发售。一期工程 2422套商铺已于去年11月成功发售，二期工程正在紧锣密鼓建设，该项目全面建成后，周口将有望成为辐射豫东南和皖西北的大型现代商贸物流中心。

　　——中原鞋都项目成功落户周口。该项目集研发、生产、展示、销售于一体，预计总投资超过200亿元，将带动1000多家制鞋企业在周口安营扎寨。届时，周口将有望成为全国最大的鞋业研发和生产基地。

　　——高等学校招生成绩斐然。全市普通高招本科上线率达到47.18%，高出全省平均水平4.2个百分点，66名考生被清华、北大和香港中文大学录取，再创历史最好水平，继续保持全省领先地位。

　　成绩来之不易，它饱含着全市各级各部门的勤奋和努力,凝聚着一千多万人民的心血和汗水，也坚定了我们奋勇前行的决心和信心。

　　过去一年，我们重点抓了以下工作：

　　（一）稳农业、强工业、兴三产，促进结构优化。二三产业占生产总值的比重达到76.9%，比上年提高1.8个百分点，产业发展层次不断提高，结构调整和转型升级取得新的进展。农业现代化水平持续提升。全面深化农村改革，稳步实施粮食生产核心示范区建设，新建高标准粮田137.2万亩，农田水利设施得到加强，粮食综合生产能力进一步提高。加快农业科技创新，周麦28通过国审，周麦系列创造了千亩方、百亩方的全国高产纪录。农业产业化取得新进展，认定省级农业产业化集群13家，新增农民合作社2085家，注册家庭农场1371家，土地流转总面积达到246.7万亩。农业内部结构进一步优化，畜牧业总产值达到210.9亿元，居全省第2位。工业发展步伐更加稳健。工业对经济的支撑作用进一步增强，规模以上工业实现主营业务收入增长15.3%；利润总额增长15.2%，工业对经济增长的贡献率达到52.3%。产业集群集聚效应更加明显，全市产业集聚区完成固定资产投资增长22%；沈丘产业集聚区晋级“河南省二星级产业集聚区”，项城等7个产业集聚区晋级“河南省一星级产业集聚区”，西华县产业集聚区被认定为“省级经济技术开发区”，食品加工、纺织服装、医药化工、装备制造、新型建材、电缆电气6大主导产业集群培育持续推进。企业科技创新能力不断增强，7家企业被认定为高新技术企业。服务业成长速度明显加快。服务业完成增加值506.5亿元，增长10.5%，创近年来最好水平。城乡市场保持繁荣，仓储物流、教育培训、信息和科技服务等生产性服务业成长迅速，文化旅游、住宿餐饮、客运邮政等生活性服务业保持活力。5个商务中心区、5个特色商业区全部开工建设，郸城县商务中心区被省政府授予“2014年度河南省十快商务中心区”称号，服务业发展载体进一步拓展。房地产业保持健康发展态势，完成开发投资209.9亿元、销售收入148.2亿元。旅游业稳步发展，全市接待游客2078万人次，旅游总收入增长16%。金融业运行平稳，银行业金融机构新增存贷比53.6%，同比增加28个百分点。与省国土资源开发投资管理中心、省农发行和省水利投资公司签署战略合作协议，融资渠道不断拓宽。

　　（二）扩总量、提质量、建机制，突出项目建设。建立中心城区重点项目协调推进周例会、县市重点项目协调推进月例会制度，把控项目推进质量和速度，保持投资对经济增长的拉动作用。182个市重点项目完成投资823.2亿元，占年度计划的122%；“双十五”工程和48个中心城区“四个一批”项目超额完成年度计划。一批重大项目建设进展顺利，鸿闽纺织、圣光医药医疗器械、金丝猴高档巧克力生产等项目超额完成年度投资计划，沙颍河水系城区段综合治理一期工程、碧桂园城市综合体项目有序推进，南水北调工程（周口段）基本完工。全市经济发展的后劲持续增强。

　　（三）打基础、抓管理、塑形象，改善城乡面貌。全面落实《加快中心城区发展意见》，中心城区建设管理水平进一步提高。周口市城市总体规划纲要通过专家评审，港口物流产业集聚区、商务中心区等一批规划编制完成并付诸实施，和谐公园、儿童医院建成投用，中州大道改造完成，连接老城区和经济开发区的八一路、五一路、汉阳路全面打通，东外环路（颍东路段）全线贯通，连接东外环路的沙颍河大桥基本建成，文昌大道升级改造工程顺利进行，新周高速连接线启动前期工作，一批污水管网及泵站改造完成，东区南水北调水厂开工建设，中心城区综合承载能力得到提升。持续开展省级文明城市创建活动，积极探索加强城市管理的新办法，综合治理城市“脏、乱、差、堵”和“两违”现象，市容市貌有所改善。周西快速通道建成通车，周项、郸沈快速通道和郸淮一级公路建设稳步实施，城市之间基础设施互联互通水平进一步提高。支持和引导各个县城科学规划建设，8个县城均表现出中小城市发展的较强活力。城镇化率达到36.19%，比上年提高1.41个百分点。全面实施农村公路“三年行动计划”，新建、改建农村公路1626公里，完成农网改造升级工程，继续推进光缆通乡、通村工程，农村生产生活和发展条件明显改善，城乡一体化发展迈出新步伐。

　　（四）增活力、添动力、引外力，深化改革开放。以推动重点领域改革为抓手，深化行政审批制度改革，减少市级行政审批事项56项；认真落实工商登记制度改革和“营改增”试点，尊重和鼓励各县市区改革创新，全社会加快发展的活力和动力进一步释放。继续把招商引资和承接产业转移作为战略举措，新签约招商引资项目265个，总投资1064.8亿元；新开工亿元以上项目152个，到位资金156.8亿元；合同履约率、项目开工率和资金到位率均居全省前列。总投资130亿元的七一路东段棚户区改造、总投资67亿元的领导者国际汽车文化园、投资30亿元的美国好时金丝猴巧克力生产基地和投资25亿元的红星美凯龙生活广场等一批龙头型、基地型项目成功签约，开放招商的质量持续提升，承接产业转移的规模持续扩大。外贸进出口累计完成7.92亿美元，其中出口7.13亿美元，增长28.4%；新批外商投资企业7家，实际利用外资4.68亿美元，对外经济合作取得新成效。

　　（五）重建设、严治理、减排放，保护生态环境。大力推进蓝天、碧水、乡村清洁“三大工程”建设，环境承载能力持续提升，我市被省政府表彰为环境保护先进单位。清洁能源改造稳步实施，秸秆焚烧得到有效遏制，淘汰黄标车和老旧机动车1.35万辆，优良空气质量天数达到257天。5个地表水质断面监测达标率85%，高于省定责任目标15个百分点，城市集中饮用水源地取水水质达标率100%。34个“乡村清洁工程”项目实施方案通过评审，3个省级生态镇、12个省级生态村、143个市级生态村创建通过验收。完成造林面积11.7万亩，城市森林工程新增绿地18.2万平方米，森林覆盖率达到8.8%，较上年提高0.35个百分点，全国绿化模范城市创建取得新进展，保持了生态竞争力的传统优势。

　　（六）保就业、惠民生、增福祉，推动社会进步。积极实施“十项重点民生工程”，认真做好就业、社保、保障房建设等工作，新增城镇就业7.6万人，城镇登记失业率控制在4%以内，企业退休人员基本养老金继续提高，保障性住房建设任务完成率达到115.7%，住房公积金覆盖率较上年提高13.9个百分点。中心城区“三校两园”和县市一批新建、扩建学校项目进展顺利，新招聘教师4372人，教育均衡发展水平进一步提高。推动职业教育健康发展，教育教学质量明显提升。广播电视塔建成投用，文化惠民活动深入推进，举办广场专场文艺晚会500多场，农村电影放映工程完成5万多场，豫剧《山城母亲》和《梨园风流》获第九届“全国戏剧文化奖”，越调历史剧《大明朱元璋》和豫剧现代戏《口上的女人》获河南省第十三届戏剧“文华大奖”。成功举办了第五届中国（淮阳）非物质文化遗产展演。文明单位、文明乡村建设取得实效。全民健身运动广泛开展。成功申办河南省第十三届运动会。县级公立医院改革全面启动，民营医院发展势头良好，医疗卫生服务体系日臻完善。出生人口素质持续提高，人口自然增长率控制在6％。以内。300张床位的市老年护理院一期工程建设完成。美丽乡村建设成效明显，农村人居环境有所改善。扶贫开发取得新的进展，20万人实现稳定脱贫。又有101万农村居民和农村师生饮水安全问题得到解决。中心城区“10件民生实事”全面完成，新增公交车30台，新汽车客运总站建成投用，老水厂和老城区供水管网改造扎实推进，主干道实现了机械化保洁，10处“两小市场”、10座公厕、8座垃圾中转站已投入使用，有效改善了市民生活条件。严格落实安全生产责任制，重特大事故得到遏制，食品药品安全形势稳中向好。创新社会治理和群众工作机制，信访形势总体平稳，平安建设持续推进，重大案件发生数量明显下降，群众安全感和满意度大幅提升，社会大局保持和谐稳定。老龄、妇女儿童、慈善等各项社会事业全面发展。

　　一年来，市人民政府办理人大代表建议110件、政协提案338件，办结率100%。行政服务中心绿色通道、并联审批机制不断完善，推进“一站式”服务和阳光作业，审批效率进一步提高，限时办结率达到100%。深入开展党的群众路线教育实践活动，严格落实中央“八项规定”和省委、省政府20条意见，自觉履行“一岗双责”，政府自身建设和廉政工作取得新的成效。积极支持驻周解放军和武警部队建设，落实军人抚恤优待政策，做好军队转业干部和退役士兵就业安置、民兵预备役、人民防空工作，军政军民团结进一步增强。统计、审计、供销、物价、工商、海关、民族、宗教、史志、对台、外事、侨务、哲学社会科学、防震减灾和对口援疆等各方面工作都取得了新的成绩。

　　各位代表！

　　上述成绩的取得，是省委、省政府和市委科学决策、正确领导的结果，是市人大及其常委会依法监督、大力支持的结果，是市政协建言献策、积极帮助的结果，是全市上下同心同德、奋力拼搏的结果。在此，我代表市人民政府，向工作在各条战线上的工人、农民、知识分子、干部、人民解放军指战员、武警消防官兵、公安民警致以崇高的敬意！向所有关心、支持周口发展的中央和省驻周单位、社会各界人士、海内外朋友，表示诚挚的感谢！

　　各位代表！

　　在看到成绩的同时，我们也清醒地认识到，虽然我市主要经济指标增速高于全国、全省平均水平，但是生产总值与去年人代会通过的增长9.5%的目标仍有一定差距，经济社会发展相对落后的状况仍未根本改变。一是经济结构不优、产业层次不高的现状依然没有根本改变。二、三产业占生产总值的比重，特别是第三产业所占的比重与全国、全省平均水平差距很大；高新技术产业规模小，科技创新能力不强，人才储备缺乏，对新技术、新产品、新业态的引进和运用反应不够灵敏，经济发展的动力仍显单一和不足。二是城镇化水平不高，中心城区首位度低的现状依然没有根本改变。城镇化发展速度和质量低于全省平均水平，城市对农村的拉动作用不强。尤其是中心城区生产总值仅占全市生产总值的9.8%，财政收入结构也不尽合理，税收收入占公共财政收入比重较低，缺少主要的税源支撑，中心城区不大、不强、辐射带动能力弱的“小马拉大车”现状没有明显改观；城市管理水平不高，“脏、乱、差、堵”的现状没有根本好转，市民意见还很大。三是公共服务能力不强，公共产品不能满足人民群众要求的现状依然没有根本改变。教育、医疗等与百姓生活息息相关的民生领域欠账较多，学校“大班额”和医院“住院难”问题还不同程度存在，与群众的要求还有很大差距；道路交通、安全生产、食品药品安全等领域还存在薄弱环节，土地征迁、房屋征管、环境污染方面还存在群众反映强烈的问题。四是推动发展的能力不足，人才相对缺乏的现状依然没有根本改变。部分政府工作人员不能用改革的办法解决发展中的问题，思想观念和能力素质与发展需求还有很大差距；具有新理念、新知识的高端城市规划建设管理人才，推进新型工业化、信息化和现代服务业、现代农业的专业人才严重不足，“能力恐慌”问题仍较严重。五是发展环境还不够宽松，行政效率偏低的现状依然没有根本改变。为官不为等问题在一些政府部门中不同程度存在，个别工作人员群众观念淡薄，法治观念缺乏，服务意识较差，有的有权就任性，还有的在其位不谋其政，没有好处不作为、遇到责任慢作为、给了好处乱作为，懒政怠政现象时有发生。

　　各位代表！

　　存在的问题就是我们努力改正的方向，薄弱的环节就是我们突破的重点，人民的满意就是我们工作的目标。我们将正视问题，直面困难，不掩饰，不回避，勇于担当，履职尽责，决不辜负一千多万周口人民的期望和重托。

　　二、2015年工作总体要求和目标任务

　　今年是全面完成“十二五”规划的收官之年，也是深入实施周口崛起方略、谋划“十三五”发展、加快追赶跨越的关键之年。在全国经济发展进入新常态的大背景下，我们既要看到和正视问题，更要看到机遇和有利因素；既要正确认识新常态，积极适应新常态，主动引领新常态，更要准确把握市情，努力把经济发展新常态转化为发展新机遇。综合分析各种因素，我们仍有很多加快发展的有利条件和优势。一是宏观经济环境仍然有利，我们仍然可以大有作为。中央继续把“稳中求进”确定为全国经济工作总基调，继续实施稳健的货币政策和积极的财政政策，把“努力保持经济稳定增长”放在2015年经济工作的首位；省委、省政府坚持把发展作为第一要务，继续坚持“调中求进”的经济工作总基调，强调“河南仍处于可以大有作为的战略机遇期”。中央和省委、省政府实现稳增长的坚定决心和政策措施，为我们加快发展提供了有利的宏观经济环境。二是新常态为我市经济发展提供了更多机遇，我们有加快发展的比较优势。我市农业优势突出，发展空间巨大；工业大都是与日常生活消费有关的食品、纺织、医药等加工业，没有消化重化工过剩产能的包袱；第三产业刚刚起步，发展活力持续增强。我们有一千多万人口，消费市场潜力巨大；劳动力资源丰富，交通优势明显，承接产业转移的优势突出。三是国家在重点领域的发展改革政策，为我们加快发展提供了政策机遇。中央把“加快转变农业发展方式”作为2015年五大经济任务之一，将进一步深化农村各项改革，完善强农惠农政策，推动农村土地经营权加快流转、农业规模经营水平不断提升。我市作为农业大市，将会获得更多的政策支持，全市农业发展的活力和潜力将会进一步得到释放，农业经营效益将得到进一步提升。四是我市前期谋划的一些大项目将在今年和今后一个时期陆续实施，对全市经济发展的拉动作用将会逐步显现。隆达2×66万千瓦燃煤发电、七一路东段棚户区改造、领导者国际汽车文化园和中原鞋都等大项目将全面开工。新周高速年底通车，郑合高铁下半年开工，周口融入全省发展战略、承接经济中心辐射将更加便捷。这些都充分表明，周口发展的潜力仍然巨大，后劲仍然充足。只要我们审时度势，抓住机遇，就一定能够牢牢把握发展的主动权，保持周口经济持续平稳健康发展的良好态势。

　　今年政府工作的总体要求是：全面贯彻落实党的十八大和十八届三中、四中全会精神，以及中央、省委、省政府和市委的决策部署，以邓小平理论、“三个代表”重要思想、科学发展观为指导，深入贯彻习近平总书记系列重要讲话和调研指导河南工作时的讲话精神，按照“四个全面”战略布局，深入实施周口崛起方略，主动适应和引领经济发展新常态，坚持调中求进、改中创新、转中向好、稳中有为，坚定不移地把发展作为中心任务，把改革创新作为发展动力，把新常态转化为加快发展的新优势，全面促进经济社会平稳健康持续发展，保持社会和谐稳定。

　　经济社会发展主要预期目标是：生产总值增长8%以上；一般公共预算收入增长11%；全社会固定资产投资增长16%；社会消费品零售总额增长13%；进出口总额增长8%；实际利用外商直接投资增长7%；城乡居民收入与经济增长同步；城镇新增就业6.3万人以上；居民消费价格涨幅3%左右；节能减排完成省定目标任务；发展方式转变取得新进展。

　　贯彻总体要求，实现预期目标，必须按照中央、省委、省政府和市委的决策部署，坚持做到“一个坚定不移，五个更加注重”。“一个坚定不移”，就是坚定不移地实施周口崛起方略。以周口崛起方略为引领，持续开展“九大攻坚”“十大建设”，不动摇、不懈怠、不折腾，持续求进，久久为功，打造周口“五大一强”社会形象。“五个更加注重”，一是更加注重结构调整。把调整经济结构作为主动适应新常态的关键抓手，努力做强工业、做大服务业、做优农业，促进经济转型升级、提质增效。二是更加注重项目导向。坚持工作项目化、项目目标化、目标责任化、责任考评化，注重把各项工作当成项目去策划、去实施、去考核，确保工作有实招、干部出实力、发展见实效。三是更加注重改革创新。用改革的精神、改革的思路和改革的办法破解发展难题，加快转变职能，激发市场活力；强化创新驱动，鼓励开展产品创新、业态创新、商业模式创新，推动大众创业和万众创新，积极培育新的经济增长点，激发全社会发展活力。四是更加注重环境保护。全面实施新修订的环境保护法，坚持源头严防、过程严管、后果严惩，持之以恒推进节能减排和生态环境保护，努力让美丽与发展同行，保持周口在生态环境方面的传统优势。五是更加注重民生改善。始终把保障和改善民生作为一切工作的出发点和落脚点，持续实施和办好一批与老百姓生活密切相关的民生实事，让人民群众共享改革发展成果。

　　各位代表！

　　发展不容停歇，使命不容懈怠。我们一定把目标牢记心中，把责任扛在肩上，克难攻坚，埋头苦干，凝神聚力促发展，尽心竭力惠民生，奋力谱写周口崛起新篇章。

　　三、2015年重点工作

　　今年，我们将着力抓好十个方面的工作。具体来说，就是切实做到10个“持续推进”，努力实现10个“进一步提升”。

　　（一）持续推进重点项目建设，进一步提升经济发展后劲。继续把项目建设作为加快发展最直接、最有效的载体，优化项目结构，提升项目质量，扩大投资总量，为长远发展蓄积后劲。

　　加快推进重点项目建设。突出抓好以“双十五”和“四个一批” 为核心的193个重点项目，切实提高项目资金到位率、开工建设率和建成投用率。扎实推进隆达2×66万千瓦燃煤发电、南京熊猫冰柜、圣光医药医疗器械、盛鸿纺织等十五个工业项目，欧蓓莎国际商城、玉文化产业园、通用机场、陆鼎国际商贸城等十五个基础设施项目，强化“双十五”工程对项目建设的引领作用。加快推进华耀商贸城二期、中原鞋都建设，西奥电梯、红鑫滚动体生产，周口西500千伏变电站、港口码头一期工程、七一路东段和西城棚户区综合改造等 “四个一批”项目，保持中心城区快速发展势头。建成新周高速公路、郸沈快速通道、郸淮一级公路；积极推进郑合高铁、禹亳铁路建设；启动实施新增省道324（文昌大道向东延伸工程）一级公路建设项目；切实抓好一批农业和水利基础设施项目，实现重点项目在经济领域的全覆盖。

　　加强项目谋划和推介。抓住国家、省在水利工程、中西部铁路等方面加大投资力度的政策机遇，使更多项目纳入上级规划建设范围。积极谋划濮阳经周口至潢川铁路、宿州经周口至漯河铁路、贾鲁河通航工程等项目，争取列入省和国家“十三五”规划；争取周口经驻马店至南阳高速公路建设项目早日开工，加快沙颍河周口至省界航道升级改造项目推进步伐。继续谋划一批产业发展项目、基础设施项目、环境保护项目、社会民生项目，做好项目储备，为经济社会发展提供后续动力。

　　继续完善项目推进机制。坚持并完善中心城区重点项目协调推进周例会、县市重点项目协调推进月例会制度，适时组织项目集中开工。不断完善项目联审联批机制，加快审批权限下放，定期进行项目观摩点评。建立项目建设管理台账，加强项目督导检查，卡紧项目推进时间节点，提高项目建设的速度和质量。

　　（二）持续推进农村改革，进一步提升农业现代化水平。全面深化农村改革，加快推进农业发展方式转变，塑造“大农业”形象，努力建设现代农业强市。

　　继续实施高标准粮田“百千万”工程。围绕粮食生产核心示范区建设，今年再建设高标准粮田135万亩。深入开展“红旗渠精神杯”竞赛，推进农田水利基础设施建设。不断提升农业机械化装备水平，力争农业机械化率达到80%以上。加大现代农业生产技术和良种良法的推广应用，不断强化农业科技支撑，进一步增强粮食综合生产能力，保持产粮大市地位。

　　着力推进农业产业化集群培育工程。推进农业与工业、服务业的高度融合，注重周口农业品牌塑造，促进农业集群化发展。加快国家农业综合标准化示范市建设，加强农业投入品管理和产品质量检测，保证农产品质量安全，全面提升农产品的品质和信誉。坚持以产业集聚的理念发展蔬菜产业，大力支持扶沟与北京新发地及“一亩田”集团电商的合作，创新“互联网＋蔬菜”的发展模式。推进标准化、规模化、生态化养殖场（区）建设，新建、改扩建大中型标准化养殖场（小区）180家以上。大力发展林业经济，实现生态效益和经济效益同步增长。

　　鼓励发展新型农业生产经营方式。积极发展多种形式适度规模经营，提高农业经营组织化程度，扶持农民专业合作社及新型经营主体发展，稳步推进供销体制改革，增强农业抗御市场风险能力。加快建立规范有序的土地流转市场，释放土地流转潜力。加快发展都市生态农业，规划建设一批现代都市农业示范园区。

　　加大扶贫开发攻坚力度。继续推进精准扶贫，将有限的扶贫资金真正用到最需要帮助的农户和个人。用好大别山连片扶贫开发的政策机遇，以整乡推进和示范村建设为重点，完成200个贫困村整村推进任务，再让20万农村贫困人口实现稳定脱贫。

　　持续改善农村生产生活条件。切实抓好美丽乡村创建，加快改善人居环境，提高农民素质，推动“物的新农村”和“人的新农村”建设统筹并进。实施好“农村公路三年行动计划”，建成农村公路1100公里。落实好农村饮水安全工程建设，再让108万群众和师生吃上安全水。

　　（三）持续推进生产要素集聚，进一步提升工业质量效益。坚定不移实施“工业兴市”战略，持续扩总量、优结构、促集聚、提效益，确保工业发展质量再有较大提升。

　　大力推进产业集群培育。以加快产业集聚区建设攻坚为引领，继续抓好产业集聚区基础设施建设，逐步完善生产性服务业配套。建立健全产业集聚区企业准入和退出机制，提高企业入驻产业集聚区的技术、环保、投资强度、税收等标准，淘汰落后产能，清退低就业、低效益企业和闲置土地企业，切实提高单位土地产出效益。坚定不移实施食品加工、纺织服装两个千亿元，医药化工一个五百亿元，电缆电气、装备制造、新型建材三个三百亿元主导产业集群的培育，抓引导、抓保障，确保集群“强筋健骨”，早成体系。

　　重点支持企业转型升级。积极促进重点企业开展技术改造，提高自主创新能力，推进企业智能转型。持续优化产业结构和产品结构，加快培育大企业、大集团、大品牌，提高核心竞争力。支持企业上市融资，建立企业上市、企业挂牌工作长效推进机制，提高企业上市直接融资的积极性。继续开展市长质量奖评选工作，推进质量立市。

　　积极推进工业化和信息化融合。主动承接“互联网＋”行动计划，以信息化引领工业化，加快推进工业重点行业数字化、智能化，促进传统产业高端化、新兴产业规模化。加快“智慧城市”“智慧企业”和中小企业信息服务平台建设，积极引导电商企业与传统企业对接，鼓励引导本地企业建立电商平台，打造网上产业集聚区，实现产品上线交易。

　　突出抓好中心城区工业发展。坚持并不断完善中心城区工业经济每月推进例会制度，建立中心城区工业统计分析平台。市经济开发区、高新技术产业开发区、港口物流产业集聚区，分别围绕各自主导产业，努力在产业规模壮大、产业链条拉长、产业层次提升方面实现突破，持续把中心城区建成以农副产品精深加工、机械装备制造、电缆电气、物流加工为特色的产业基地，挺起中心城区工业的“脊梁”。

　　不断深化企业服务。完善产销对接、银企对接、用工对接和培训教育机制，搭建产销协调、银企合作、人才服务等平台，支持企业发展。着力解决中小微企业融资难、融资贵问题。加大对企业“三乱”行为查处力度，进一步清理和规范涉企收费项目，落实各项扶持政策，不断优化企业发展环境。

　　（四）持续推进新兴产业培育，进一步提升服务业成长速度。把加快服务业发展作为优化经济结构的关键抓手，丰富业态，提升层次，促进服务业发展提速、比重提高、水平提升。

　　加快商务中心区和特色商业区建设。推进产业招商和培育，以高端百货、休闲娱乐、商务服务、住宿餐饮等行业为重点，积极引进城市综合体、特色街、名家名店、星级酒店、电商平台等项目，加快集聚产业和人气，推动“两区”成为新的经济增长点，力争早日成为促进服务业发展的综合平台。

　　做大做强高成长性服务业。抓好一批物流、商贸业重点项目，推动华耀商贸城全面接替荷花市场功能，形成新的商贸物流繁荣核心区，奠定建设“大商贸”的基础。加快红星美凯龙生活广场项目建设，推动形成高档建材、家具交易物流中心。继续支持农产品专业交易市场建设，争取有更多专业市场进入国家农产品流通骨干网络。继续抓好羲皇故都、关帝庙等景区提档升级，丰富旅游景点，做大旅游产业。优化金融生态环境，支持银行、证券、保险、信托、基金等各类金融机构在周口注册和经营发展，不断丰富金融业态。积极发展电子商务、第三方物流、开发设计、检验检测等生产性服务业，保持服务业快速成长活力。

　　提升改造传统服务业。全面落实《促进房地产市场平稳健康发展的意见》，保持房地产经济发展活力。优化城乡商业网点布局，支持大型商贸企业建设网上商城，拓展农村经营网络，鼓励中小微企业开设网店。以家政服务试点城市建设为平台，面向城乡居民，加快发展家政服务等新兴服务业。积极发掘培育周口特色餐饮品牌和“老字号”企业，发展适合大众消费的住宿餐饮、生态休闲、演艺娱乐、文化体验服务设施。加强对物业管理企业的监管，规范物业管理市场，提升物业管理水平。

　　（五）持续推进中心城区发展，进一步提升城市整体形象。坚持实施中心城区带动战略，切实提高城市建设管理水平，增强城市综合承载和辐射带动能力，构建新型城镇体系。

　　加快中心城区建设步伐。切实增强规划意识，用好规划成果，有序推进城市建设、完善城市功能、提高城市品位。以“十大建设”为重点，加强中心城区基础设施建设。继续完善城市路网，完成南六路、南八路建设，尽快形成新火车站、汽车站环线交通路网；东外环路新建工程实现全线贯通，改造提升太清路，建成新周高速公路连接线，打通川汇大道，实施文昌大道和城市出入口改造提升、庆丰街延伸、开元大道东通港区等工程，实现市区道路互联互通。继续完善城市交通管理基础设施，提高交通管理技术装备和应用水平。集中精力办好 “中心城区10件民生实事”，完善城市公交体系，开工建设华耀城汽车客运站；新增公交车辆20台，增开通往火车站、华耀商贸城等重要区域的公交班线；新建和改造老城区5条道路，完成7条道路路灯提升改造；新建、改建5个居民健身广场（园）；加快老水厂和城市供水管网改造，加快东区南水北调水厂建设，提高供水能力和供水质量，让广大市民早日用上干净卫生的丹江水；加快城市排水管网改造建设，提高城市排水和污水处理能力；加快流沙河、洼冲沟、运粮河生态水系治理，改善人居环境；继续建设一批新的“两小市场”、公厕、垃圾中转站、停车场，不断满足市民生活需求。

　　扎实推进城市管理攻坚。深化改革，理顺体制，健全机制，增加投入，重点解决好城市管理中的缺失缺位问题。全方位加大综合管治力度，完善中心城区市、区、办、社区四级管理网络，实现责、权、利的有机统一，促进城市管理上水平。坚持中心城区城管委每月例会制度，深入开展“脏、乱、差、堵”专项治理，保持严厉打击“两违”的高压态势，持续强化城市居民的规则意识、法律意识、文明意识。规划实施精品街区建设工程，分别在城乡一体化示范区、老城区、开发区选择3个街区和若干道路，加强精细化建设、管理和维护，建成一批严管街、示范路、高品质社区，打造城市管理的精品工程和示范亮点。重点解决好群众反映强烈的垃圾乱倾乱倒、乱堆乱放和垃圾围城问题，依法对渣土车超载抛洒和三轮车非法营运进行专项治理，不断改善环境卫生和城市秩序，努力实现中心城区路畅、街净、树绿、灯亮。

　　全面加强城乡一体化示范区建设。以建设“大城区”为目标，按照“三区一中心”的功能定位，坚持规划引领、板块启动、基础突破、项目支撑，持续打造城乡一体化发展先行区、对外开放示范区、现代生态宜居区和区域商贸物流中心。加快综合服务功能区项目组装，力争早出形象、早聚人气。加快推进沙颍河城区段治理项目，形成新的城市景观。加快港区“九路四桥”和码头建设，打造新的发展极。有计划、有目标地收储一批条件较好的地块，做大土地储备总量，优先保障示范区建设用地需求。不断推进城乡基本公共服务均等化，努力建设生产、生活、环境统筹兼顾，经济、宜居、生态功能有机组合的功能复合型城市新区。

　　加快构建新型城镇体系。围绕“一中心四组团”的发展格局，引导中心城区与四个组团县市之间在产业发展、基础设施、公共服务方面加强对接，继续抓好周项快速通道建设，推进周商城区道路互联互通，方便中心城区与组团城市之间的商务和人员交流。加强路网、电网、气网、通信网建设，持续提高“组团县城”与“支撑县城”之间的基础设施连通水平。围绕郑合高铁建设，谋划好站点设置及周边的开发建设，促进形成新的城市亮点。继续支持鼓励各个县城加快发展，按照现代中等城市功能要求，坚持 “一基本两牵动”，加快基础设施建设，拉大县城框架。加强重点镇和新农村建设，在每个县选择1～2个产业基础好的重点镇，因地制宜，分类指导，培育特色鲜明的中心镇，推进形成有利于城乡一体化发展的新型城镇体系。

　　（六）持续推进对外开放，进一步提升招商引资实效。坚持实施开放带动战略不动摇，优化招商营商环境，主动开展产业对接，积极承接产业转移。

　　加大开放招商力度。突出企业招商主体地位，创新招商方式，明确招商重点，提高招商实效。继续以产业集聚为目标，围绕主导产业、新兴产业和服务业，编制产业发展链条招商图谱，扩大招商信息发布，紧盯资本和产业转移动态，争取引进一批投资规模大、带动作用强的工业、商业项目。加大招商项目的甄别力度，按照“宁缺毋滥”的原则，对以圈地为目的的项目坚决进行清理，不断提升招商引资和承接产业转移的实效。加快“河南省制鞋产业出口基地”和“河南省食品调味品出口基地”建设，争取设立 “河南省纺织服饰出口基地”，承接好广东鞋业等产业的成批次转移，增强外贸出口能力。

　　优化开放招商环境。积极探索新的招商途径，促进由依靠优惠政策招商向依靠自身优势和优质服务招商转变，清除各类影响项目落地的障碍，为各类来周投资者提供周到、便捷的服务。完善开放平台建设，继续提升扶沟公共保税仓库和出口监管仓库运行效率，推广“多点报关、口岸验放”通关一体化模式，探索周口海关、漯河商检合作机制，出台支持和服务我市进出口企业通关便利的一系列措施，不断优化开放招商环境。

　　（七）持续推进改革创新，进一步提升经济社会发展活力。按照中央、省和市委决策部署，继续实施一批看得准、有条件、有权限的改革，强化创新驱动，激发发展活力。

　　加快重点领域和关键环节改革。扎实推进政府职能转变，继续减少和取消审批事项，优化简化审批流程，逐步推行权力清单、责任清单和网上并联审批等改革。完成市县行政体制改革。深入落实户籍制度及各项配套改革举措。深化预算管理改革，推进预决算公开。做好“营改增”、消费税等税制改革。加强政府性债务管理。完成市投资集团组建。深入推进新一轮食品药品监管体制改革，建立全市统一的食品药品检验检测机构。完成交通综合执法体制改革。继续深化国企改革。以市县两级为重点，加快不动产登记制度改革。加大各项改革措施落实力度，让人民群众对改革成果有更多“获得感”。

　　鼓励推动大众创业。坚决落实国家有关鼓励创业的优惠政策，以创业带动就业，让各类自主创业、自谋职业人员享受到国家规定的各类费用减免，推动中小微企业健康快速发展。不断简化注册资本登记，逐步实现“三证合一”，充分提供创业便利，激发社会创业活力。周口人民勤劳智慧，蕴藏着巨大的创造潜力。政府决心为老百姓搞好创业服务，让千千万万怀揣致富梦想的人，放心大胆地进入创造财富的市场舞台，共同汇聚周口崛起的巨大动能，让周口经济始终充满勃勃生机。

　　注重创新驱动发展。优化促进科技创新的政策环境，加强知识产权保护，形成万众创新的良好局面。大力推进周口高新区建设，研究制定促进高新区发展的政策措施，支持新材料、光机电一体化、先进装备制造等特色产业发展，不断提高园区主导产业的整体质量和技术水平。大力支持周口师范学院建设大学科技园区，孵化高成长性、高新技术产业。加强企业技术创新能力建设，力争认定1至2个省级技术创新示范企业。着力推进高新技术产业发展，新建高新技术企业5家。鼓励有条件的企业创办技术中心，支持申报各级工程技术研究中心和重点实验室，力争新建省级以上工程技术研究中心3家。围绕主导产业和特色产业，积极组织开展科技联合攻关和成果展示、对接、洽谈等活动，促进更多的科技成果在我市开花结果。

　　提高要素保障能力。严格落实耕地保护责任，划定永久性基本农田，管好用好各类建设用地指标，完成省下达的征地率、供地率责任目标。坚持集约节约，切实保障市级以上重点项目建设用地。积极稳妥推进农村土地综合整治试点、人地挂钩试点，挖掘土地资源潜力。加强银企对接，研究政银担企四方合作新举措，破解小微企业抵质押不足瓶颈。改革投融资体制，鼓励民间资本发起设立创业投资基金和股权投资基金，推行政府与社会资本合作模式，吸引更多社会资本投入城市基础设施建设和运营。探索金融服务“三农”新模式，提高农村金融服务能力和水平。推进市政府与省国土资源开发投资管理中心、省水利投资公司、省农业发展银行、中原银行、平安银行的战略合作，用好省政府3支新型城镇化发展母基金创造的政策红利，筹划设立中心城区及各县城的子基金，服务城市发展。落实《加快中心城区发展引进高层次和紧缺人才办法（试行）》，积极引导人才、技术等创新要素向企业集聚，适应企业发展需求。

　　（八）持续推进文化繁荣，进一步提升周口文化影响力。充分发挥文化资源优势，加强文化精品创作，发展文化事业，壮大文化产业，不断提升周口文化影响力，提高周口发展软实力。

　　着力提升文明素质。以社会主义核心价值观教育为引领，加强社会诚信体系建设，以政务诚信带动商务诚信和社会诚信，形成良好的社会风尚。以开展“出彩周口人”系列报道活动为契机，讲好周口故事，展示周口形象。加强法制宣传教育，提高全民法律素养。加强公民道德建设，扎实开展“做文明人、办文明事”主题实践活动，提高市民文明素质。

　　支持文化精品创作。加大对优秀文化艺术作品、优秀社会科学成果的奖励力度，鼓励引导广大文艺工作者、哲学社会科学工作者扎根基层、扎根人民，创作出更多优秀作品。支持文化院团建设，加强业务指导和绩效考核，推进形成优秀作品创作基地。注重加强文化创作队伍培养，培育一批德艺双馨的名作家、名记者、名学者、名艺术家，保持文化创造活力，扩大周口外在影响。

　　切实搞好文化惠民。加快构建现代公共文化服务体系，保证人民群众享受基本公共文化服务。健全市文化艺术中心、县级两馆、乡镇综合文化站等公共文化服务设施，落实各类免费开放政策。倡导全民阅读，加强知识传播，建设书香社会。积极做好文物保护和利用工作。开展公益性文化活动，丰富群众精神文化生活。开展“扫黄打非”，维护文化安全。积极实施全民健身国家战略，开工建设市游泳馆和老年健身馆，完善市体育中心功能，促进全民健身活动。做好省第十三届运动会各项筹备工作，推动群众体育和竞技体育协调发展。

　　大力发展文化产业。鼓励创办文化创意、文化传播、文化旅游企业，拓展文化产业发展领域，不断提高文化产业规模化、集约化、专业化水平。组建周口广电融媒集团，支持周口报业集团拓展经营业务，壮大业务规模，打造文化产业新亮点。支持淮阳文化改革发展实验区积极探索，力争今年取得实质性进展。

　　（九）持续推进环境治理，进一步提升生态文明程度。坚持把生态文明建设贯穿于经济社会发展全过程，以更大的决心和更有力的举措，打造天蓝、地绿、水清的美丽周口。

　　加大环境保护力度。认真贯彻执行新修订的环境保护法，实行最严格的环境评价制度。加强对建设项目、污染源、畜禽养殖、饮用水源等环境问题的监管，坚决查处环境违法行为。严格落实节能减排总量预算管理机制，着力实施一批循环经济、清洁生产和节能降耗示范工程。积极倡导绿色生活方式和消费理念，引导群众积极参与生态文明建设。

　　加强生态环境治理。继续实施“蓝天工程”，加强空气质量监测治理，加快推动重点行业脱硫脱硝除尘改造，加强黄标车、老旧机动车淘汰，严禁秸秆、垃圾焚烧，切实遏制大气污染。继续实施“碧水工程”，推进沙颍河、贾鲁河等重点河流治理，加强污水排放和治理设施运行监管，确保主要河流水质稳中趋好。继续实施“乡村清洁工程”，开展以生活污水和生活垃圾治理为主要内容的农村人居环境综合整治，加大对农村规模化养殖场及养殖大户污染整治力度，促进农村生活环境持续改善。

　　继续创建全国绿化模范城市。突出中心城区绿化，全面开展花园式单位创建工作，增加绿化面积。加快实施廊道绿化，完成绿化面积3.45万亩。继续抓好农田林网建设，将农田防护林建设与高标准粮田“百千万”工程建设相结合，完成农田林网5.7万亩，农田林网控制率达到94%以上。

　　（十）持续推进社会事业发展，进一步提升人民生活质量。以“十项重点民生工程”为抓手，扎实做好民生工作，不断提高人民群众生活质量。

　　扎实做好就业和社会保障。继续实施全民技能振兴工程和以创业带动就业工程，构建更加完善的公共就业服务体系，新增城镇就业6.3万人以上，新增农村劳动力转移就业10万人以上，城镇登记失业率控制在4%以内。全面启动机关事业单位养老保险改革工作，继续提高城乡低保、农村五保供养和优抚对象待遇，不断完善城乡社会救助体系，兜住保障困难群众基本生活底线。探索社区养老新模式，在中心城区建设5个以上社区养老服务示范点。加大保障性住房建设力度，开工建设保障性住房2.2万套，改造农村危房1.5万户，扩大住房公积金覆盖面。

　　加快发展教育卫生事业。以做强“大教育”为总抓手，坚持义务教育区域均衡发展，大力增加城镇教育资源，全面改善农村办学条件；实施第二期学前教育三年行动计划，扩大公办幼儿园和普惠性民办幼儿园覆盖率。把振兴中心城区教育放在重中之重的位置，整体提高中心城区各级各类学校办学水平，加大周口一高争创中原名校力度，新建和改扩建2校5园，逐步缓解中心城区“大班额”“入园难”问题；新建1所特殊教育学校，改善残疾学生就学条件；深入实施职业教育攻坚二期工程，支持周口科技职业学院晋升本科，扎实推进联营职专、周口幼师晋升专科，推进周口职业技术学院、职教中心（技工学校）加快发展，支持周口师范学院向综合性应用型大学发展，提升人力资源开发能力。积极推进县级公立医院综合改革。大力发展社会办医。完善城乡居民基本医保，全面实施新农合大病保险制度。加强重大疾病防控，积极发展中医药事业。加快市中心医院东新区医院、市中医院东新区医院、市老年护理院二期工程建设进度，加强基层公共医疗卫生服务体系建设，持续推进基本公共卫生服务均等化，着力解决人民群众“看病难”“看病贵”问题。扎实做好人口和计划生育工作，促进人口长期均衡发展。

　　切实维护社会稳定。树立“大平安”理念，着眼“双安”，推进“双治”，强化“双基”，促进社会治理现代化。建立健全化解各类风险的体制机制，抓好阳光信访、积案化解等工作，维护信访稳定。加强社会治安防控体系建设，严厉打击各类刑事犯罪，继续开展平安周口建设攻坚，维护社会大局和谐稳定。坚决打击非法集资，防范和化解金融风险。全面推进“依法治安”，强化责任体系建设，坚决防范和遏制重特大安全事故发生。严格落实食品药品生产经营企业的主体责任，严厉打击非法生产、销售行为，保障人民群众饮食用药安全。

　　继续开展双拥共建，积极创建双拥模范城，支持驻周解放军、武警部队、预备役部队、民兵和人民防空建设，促进军民融合式发展。维护农民工、妇女儿童合法权益，关心残疾人事业。加强民族宗教工作，促进民族经济发展和民族关系和谐。支持工会、共青团、妇联、工商联等人民团体在经济社会发展中发挥桥梁纽带作用。继续做好统计、审计、物价、海关、气象、史志、外事、侨务、防震减灾、应急救援、对口援疆等工作。

　　四、勇于担当，履职尽责，努力建设人民满意的政府

　　人民的满意是对政府的最高奖赏，建设人民满意的政府，是我们坚持不懈的追求。我们将持续弘扬勇于担当、敢于负责的精神，讲政治、顾大局，善作为、重实干，努力在更高水平上建设人民满意的政府。

　　坚持党的领导，维护大局。市委是全市各项工作的领导核心。坚持市委统一领导，维护市委权威，保持全市上下团结统一，是做好一切工作的根本保证。我们将始终维护周口发展大局，自觉执行市委各项决策部署，时刻与市委保持高度一致，共同凝聚全市发展智慧，形成发展合力，维护好全市政治经济社会发展的大好局面。

　　坚持勇于担当，敢于负责。勇于担当的实质就是敢于负责，就是想干事、不避事、干成事。我们将持续弘扬敢于负责、勇于担当的精神，始终坚持事业为重、发展第一、人民利益至上，敢于旗帜鲜明，敢于较真碰硬，保持动真格的冲劲、求突破的钻劲、见实效的拼劲、不懈怠的韧劲，困难面前不退缩，矛盾面前不躲闪，挑战面前不畏惧，敢抓敢管、敢于碰硬，不折不扣地完成既定工作目标。

　　坚持务实重干，勤政为民。务实重干是政府的职责，服务人民是政府的宗旨。我们将始终把为人民谋发展、增福祉作为庄严使命，把务实重干当做兴业之基，把勤政为民当做立身之本，坚定追赶跨越的信心，保持专注发展的定力，提升推动发展的能力，完善科学发展的措施，立说立行、善作善成。围绕提高行政效能，持续加强作风纪律建设，克服“躁假浅怨混”不良习气，对行政不作为进行专项治理，惩戒懒政怠政行为，进一步解决群众办事难问题，提高群众对政府工作的满意程度。

　　坚持依法行政，接受监督。全面依法治国是中央的战略布局，坚持依法行政是政府的基本遵循。我们将自觉做到尊法学法守法用法，切实做到法无授权不可为、法定职责必须为。坚持依法履行政府职责，健全依法决策机制，全面推进政务公开，扎实开展服务型执法示范单位创建，不断提高依法行政水平。依法接受人大及其常委会的法律和工作监督，主动接受政协民主监督，严格执行人大及其常委会的决议决定，认真办理人大代表建议和政协提案，广泛听取民主党派、工商联、无党派人士、人民团体意见，自觉接受司法监督、社会监督、舆论监督，让政府工作充分体现人民的意愿。

　　坚持廉洁从政，树好形象。作风建设永远在路上。我们将认真履行“一岗双责”，严格遵守中央“八项规定”和省委、省政府20条意见，坚持不懈纠正“四风”，巩固深化党的群众路线教育实践活动成果，打造阳光政府。严格执行党政机关厉行节约反对浪费条例，认真贯彻国务院“约法三章”，完成党政机关公务用车制度改革，确保“三公”经费和财政供养人员只减不增。严格落实廉政建设责任制和主体责任，加强行政监察和审计监督，强化廉政教育，遵守廉政准则，做到无禁区、全覆盖、零容忍，以实际行动取信于民，树立为民务实清廉的政府形象。

　　各位代表！

　　使命催人奋进，发展时不我待。推进周口崛起是我们的责任，过上美好生活是人民的期盼。我们所有的努力和奋斗，归根到底是为了让周口人民生活得更加幸福、更有尊严、更有地位。让我们坚定地团结在以习近平同志为总书记的党中央周围，在省委、省政府和市委的坚强领导下，齐心协力，团结奋斗，锐意进取，真抓实干，不断开创周口崛起、富民强市、全面建成小康社会的新局面。
